# Editorial: 15 years of Frontiers in Cellular Neuroscience: super-resolution microscopy in the healthy and the injured brain

**DOI:** 10.3389/fncel.2024.1448206

**Published:** 2024-07-09

**Authors:** Egor Dzyubenko, Jianxu Chen, Katrin I. Willig

**Affiliations:** ^1^Department of Neurology and Center for Translational Neuro- and Behavioral Sciences (C-TNBS), University Hospital Essen, Essen, Germany; ^2^Analysis of Microscopic BIOMedical Images (AMBIOM), Leibniz-Institut für Analytische Wissenschaften - ISAS - e.V., Dortmund, Germany; ^3^Cellular and Molecular Imaging in Anatomy, Institute of Theoretical Medicine, University of Augsburg, Augsburg, Germany

**Keywords:** fluorescence nanoscopy, shadow imaging, radial fluctuation, synapse, primary cilia, two-photon microscopy (2-PM), expansion microscopy

The continuous evolution of light microscopy technologies has led to the development of several superresolution methods that surpass the diffraction limit of classical fluorescence microscopy. Superresolution microscopy, combined with advanced image processing algorithms, has become essential for uncovering biological mechanisms and analyzing cellular and sub-cellular morphology in both health and disease (Fuhrmann et al., 2022; Willig, 2022; Dzyubenko et al., 2023; Zhou et al., 2024). This Research Topic explores recent advances in superresolution imaging and analysis methods, which are crucial for understanding critical biological mechanisms in the healthy and the injured brain.

Visualizing the organization of synaptic proteins requires extremely high imaging resolution. In this Research Topic, Sachs et al. introduce a method that combines expansion microscopy with Airyscan microscopy, enhancing labeling efficiency and enabling multicolour fluorescence imaging with 20–40 nm spatial resolution. Their study reveals that synaptic proteins RIM 1/2, Munc13-1, and AMPA receptors are organized into trans-synaptic nanocolumns, which are critical for effective synaptic transmission. This method provides a powerful tool for exploring the nanoscale arrangement of synaptic proteins, thereby deepening our understanding of synaptic function.

Conventional microscopy methods often face issues with targeted labeling, which can be inhomogeneous and incomplete, leading to gaps in data and analysis. The review by Inavalli et al. explores the potential of fluorescence microscopy shadow imaging to overcome these challenges. Shadow imaging offers homogeneous and exhaustive fluorescence labeling of the extracellular space, resulting in cellular structures appearing as dark silhouettes, or shadows, against a fluorescent background, thereby providing clear and comprehensive visualization. Key advantages of this method include reduced photobleaching and toxicity, as well as consistent labeling across the entire sample. Originally developed to assist patch-clamp electrophysiology, shadow imaging has since evolved and found applications across various microscopy modalities. Techniques such as super-resolution STED microscopy, two-photon microscopy, and light-sheet microscopy have all benefited from the incorporation of shadow imaging. This evolution has expanded its use beyond neuroscience, demonstrating broad potential for intravital imaging in other tissues, such as bone marrow and lymph nodes.

Obtaining three-dimensional (3D) superresolution information about dynamic processes in live cells at high speed remains challenging. In this Research Topic, Tingey et al. demonstrate how single-point edge-excitation sub-diffraction (SPEED) microscopy, combined with 2D-to-3D transformation algorithms, can achieve high temporal and sub-diffraction-limit 3D resolution in subcellular structures with rotational symmetry. The authors review how this novel approach elucidates vesicle-assisted transport mechanisms in intracellular trafficking to the primary cilium, which is critical for understanding developmental disorders. Based on the superresolution imaging data obtained with SPEED microscopy, the authors propose a comprehensive model of ciliary transport.

Despite recent technological advances, applying superresolution microscopy for deep imaging in thick specimens, such as brain sections, remains challenging. To overcome the optical limitations of deep superresolution imaging, such as the deterioration of light convergence properties, Tsutsumi et al. propose a method called 2P-SRRF, which combines super-resolution radial fluctuation analysis with two-photon microscopy. The authors demonstrate that 2P-SRRF can achieve sub-diffraction-limit spatial resolution, effectively resolving features separated by 120 nm even at significant depths. The resolution enhancement was validated at depths exceeding several hundred micrometers, showcasing high-resolution *in vivo* imaging in the fifth layer of the cerebral cortex. This makes 2P-SRRF a versatile and powerful tool for neuroscience research.

This Research Topic highlights the recent advancements in superresolution imaging techniques that shed light on critical biological mechanisms in the brain. These advancements overcome the challenges in conventional fluorescence labeling, high-speed 3D superresolution microscopy, and deep brain imaging, offering versatile applications in neuroscience research.

## Author contributions

ED: Writing – original draft, Writing – review & editing. JC: Writing – review & editing. KW: Writing – review & editing.
